# An antisense *Alu* transposon insertion/deletion polymorphism of *ALDH1A1* may functionally associate with Parkinson’s disease

**DOI:** 10.1186/s12877-022-03132-1

**Published:** 2022-05-16

**Authors:** Hui-Hui Fan, Jing Zheng, Xiao-Ya Huang, Ke-Yun Wu, Lei Cui, Hao-Jia Dong, Zhen Wang, Xiong Zhang, Jian-Hong Zhu

**Affiliations:** 1 Department of Preventive Medicine, Institute of Nutrition and Diseases, Wenzhou Medical University, Wenzhou, 325035 Zhejiang China; 2 Department of Geriatrics and Neurology, the Second Affiliated Hospital and Yuying Children’s Hospital, Wenzhou Medical University, Wenzhou, 325027 Zhejiang China; 3 Department of Neurology, Wenzhou Central Hospital, Wenzhou, Zhejiang China; 4 Department of Neurology, the First Affiliated Hospital, Wenzhou Medical University, Wenzhou, Zhejiang China

**Keywords:** *ALDH1A1*, Parkinson’s disease, Polymorphism, *Alu* element, Association

## Abstract

**Background:**

Aldehyde dehydrogenase 1 (encoded by *ALDH1A1*) has been shown to protect against Parkinson’s disease (PD) by reducing toxic metabolites of dopamine. We herein revealed an antisense *Alu* element insertion/deletion polymorphism in intron 4 of *ALDH1A1*, and hypothesized that it might play a role in PD.

**Methods:**

A Han Chinese cohort comprising 488 PD patients and 515 controls was recruited to validate the *Alu* insertion/deletion polymorphism following a previous study of tag-single nucleotide polymorphisms, where rs7043217 was shown to be significantly associated with PD. Functional analyses of the *Alu* element insertion were performed.

**Results:**

The *Alu* element of *ALDH1A1* was identified to be a variant of Yb8 subfamily and termed as Yb8c4. The antisense Yb8c4 insertion/deletion polymorphism (named asYb8c4^ins^ and asYb8c4^del^, respectively) appeared to be in a complete linkage disequilibrium with rs7043217 and was validated to be significantly associated with PD susceptibility with asYb8c4^ins^ serving as a risk allele (*P* = 0.030, OR = 1.224, 95% CI = 1.020–1.470). Multiple functional analyses including *ALDH1A1* mRNA expression in blood cells of carriers, and reporters of EGFP and luciferase showed that the asYb8c4^ins^ had a suppressive activity on gene transcription. Mechanistic explorations suggested that the asYb8c4^ins^ induced no changes in CpG methylation and mRNA splicing of *ALDH1A1* and appeared no binding of transcription factors.

**Conclusions:**

Our results consolidate an involvement of ALDH1 in PD pathogenesis. The asYb8c4 polymorphism may be a functional output of its linkage disequilibrium-linked single nucleotide polymorphisms.

**Supplementary Information:**

The online version contains supplementary material available at 10.1186/s12877-022-03132-1.

## Background

Parkinson’s disease (PD) is a common neurodegenerative disorder resulted from the progressive loss of dopaminergic (DA) neurons in the substantia nigra pars compacta. While a few genes such as *LRRK2, SNCA* and *PRKN* have been identified to be mutated and causative in familial PD, limited is known in regard with the etiology of sporadic PD. Benefiting from the genome-wide association studies and the following investigations, a number of risk loci towards PD are disclosed in positions such as *RIT2*, *SIPA1L2*, and *VPS13C* [1–3]. These genetic susceptibilities, together with environmental insults such as pesticide exposure and prior head injury, are generally believed to lead to the sporadic pathogenesis of PD [4].

Aldehyde dehydrogenase (ALDH), which includes the cytosolic ALDH1 and mitochondrion-located ALDH2, is a family of aldehyde-oxidizing enzyme and catalyzes the conversion of the dopamine metabolite 3,4-dihydroxyphenylacetaldehyde (DOPAL) into 3,4-dihydroxyphenylacetic acid in DA neurons. Accumulation of the DOPAL is toxic to the neurons [5–7]. Indeed, pesticide benomyl, which inhibits ALDH activity, leads to increased production of DOPAL and cytotoxicity to rat DA neurons [8, 9]. Inhibition of ALDH activity is also considered to be a mechanism for rotenone-induced neurotoxicity [10]. ALDH1, which is encoded by *ALDH1A1*, is highly expressed in DA neurons [11], and is noted to be severely reduced in the substantia nigra pars compacta of post-mortem PD brains [12, 13]. The reduction of *ALDH1A1* mRNA expression was previously suggested as a potential blood biomarker of PD [14]. ALDH1-positive DA neurons exhibit stronger resistance than the negative ones against α-synuclein-induced cytotoxicity in mice [15]. Double knockout of *Aldh1a1* and *Aldh2* leads to a significant loss of DA neurons in the substantia nigra of mice [16].

Our previous study of six tag-single nucleotide polymorphisms (SNPs) of *ALDH1A1* suggested that rs7043217 is significantly associated with PD risk [17]. During the study, we surprisingly observed a length polymorphism in the intron 4. We hypothesized that this length polymorphism might play a role in PD, and thus performed an association validation and multiple functional analyses.

## Methods

### Subjects

A total of 1003 Han Chinese were recruited from eastern China, including 488 sporadic PD patients (252 males and 236 females) and 515 controls (286 males and 229 females). The median age of the patients and controls was 66 (interquartile range, 59–72) and 59 (interquartile range, 51–68), respectively. PD patients were diagnosed by two neurologists according to the UK Parkinson's Disease Society Brain Bank Criteria [18]. Patients with a family history of PD, or with secondary and atypical parkinsonism were excluded. Control subjects were free of neurological disorders determined by medical history, physical and laboratory examinations. For quantitation of the *ALDH1A1* mRNA levels, another 29 control subjects were recruited, including 15 subjects carrying the insertion genotype of the length polymorphism (7 males and 8 females; median age, 66 years, interquartile range, 62–68) and 14 with the deletion genotype (7 males and 7 females; median age, 67 years, interquartile range, 64–69).

### Genotyping

Genomic DNA was extracted from peripheral blood samples using TIANamp Genomic DNA Kit (Tiangen, Beijing, China) following the manufacturer's instruction. The length polymorphism was analyzed using polymerase chain reaction (PCR) followed by agarose gel electrophoresis. Direct sequencing of the products was performed using an ABI PRISM 3730 DNA Analyzer (Applied Biosystems, Foster City, CA, USA) after processed with BigDye Terminator v3.1 kit (Applied Biosystems, Foster City, CA, USA) at the Beijing Genomics Institute (Beijing, China). The primers, as well as the primers used below, were all detailed in Additional file 1: Table S1.

### Methylation analysis

Methylation levels were analyzed by bisulfite sequencing PCR. Bisulfite conversion of genomic DNA was performed using EZ DNA Methylation-Gold kit (Zymo Research, Orange, CA, USA) according to the manufacturer’s protocol. Bisulfite-treated DNA was used for amplification of the CpG island, and the upstream and downstream CpG sites. PCR products were cloned into the plasmid pMD-19 T (Takara, Shiga, Japan) and transformed into *E*. coli. Ten colonies were randomly selected and sequenced.

### Blood cell isolation and real-time PCR

Peripheral blood mononuclear cells (PBMCs) were isolated using a human lymphocyte separation kit (TBD Science, Tianjin, China) according to the manufacturer's protocol. Total RNA was extracted using TriPure Isolation Reagent (Roche, Indianapolis, IN, USA). An amount of 500 ng RNA was used for synthesis of the first-strand cDNA by PrimeScript RT reagent Kit (Takara, Shiga, Japan). Real-time PCR samples were prepared with Faststart Essential DNA Green Master (Roche, Indianapolis, IN, USA) and amplification was performed using the CFX connect real-time system (Bio-Rad Laboratories, Hercules, CA, USA). Value of the cycle threshold was determined by automated threshold analysis using the Opticon Monitor 3.1 software (Bio-Rad Laboratories, Hercules, CA, USA).

### Plasmids and luciferase reporter

Target fragments were cloned into pMIR-Report firefly luciferase reporter (Ambion, Austin, TX, USA; between *Sac* I and *Hind* III) and pEGFP-C1 (Clontech, Palo Alto, CA, USA; between *Xho* I and *Pst* I). The constructs of firefly luciferase reporter were co-transfected with the Renilla luciferase vector pRL-TK (Promega, Madison, WI, USA) into SK-N-SH cells (Cell Bank of Chinese Academy of Sciences, Shanghai, China). Luciferase activity was determined 24 h after transfection by a Dual-Luciferase Reporter Assay kit (Promega, Madison, WI, USA) following the manufacturer’s protocol. Luciferase mRNA levels were determined 24 h after transfection by real-time PCR. Firefly luciferase data were normalized to the activity or mRNA levels of Renilla luciferase. For EGFP expression, fluorescence intensity was observed and quantified 48 h post-transfection of the EGFP constructs in SK-N-SH cells.

### DNA pull-down assay

Biotin-labelled double-stranded oligonucleotides were amplified from intron 4 of *ALDH1A1* using the primer pair listed in Additional file 1: Table S1 and then purified using MiniBEST Agarose Gel DNA Extraction Kit (Takara, Dalian, China). Nuclear fraction was extracted from SK-N-SH cells (American Type Culture Collection, Manassas, VA, USA) using Nuclear and Cytoplasmic Protein Extraction Kit (Beyotime, Shanghai, China). Five nM of the purified oligonucleotides was then incubated with 500 μg nuclear extracts in a binding buffer (25 mM Tris, 150 mM NaCl and 1 mM PMSF; pH 7.2) for 30 min at room temperature with gentle rotation, followed by 2 h incubation with the addition of 50 μL streptavidin-agarose beads (Sigma, St. Louis, MO, USA). The complexes were washed 4 times with the binding buffer at 4 °C. Proteins were eluted using a lysis buffer (60 mM Tris–HCl at pH 6.8, 5% glycerol, 2% SDS) and subjected to 10% SDS-PAGE as previously described [19]. After visualization using a silver staining kit (Beyotime, Shanghai, China), designated protein bands were cut and subjected to liquid chromatography/tandem mass spectrometry analysis (LC–MS/MS) at Shanghai Applied Protein Technology (Shanghai, China). The LC–MS/MS analysis was performed using an EASY nLC 1000 system coupled to a Q-Exactive mass spectrometer (Thermo Fisher Scientific, San Jose, CA, USA).

### Sequence and statistical analysis

Sequence similarity was searched using the BLAST tool at NCBI (http://www.ncbi.nlm.nih.gov/blast). Gene Ontology analysis was performed at http://geneontology.org/. Statistical analyses were performed using the Statistical Package for Social Science Program (SPSS for Windows, version 23.0). Hardy–Weinberg equilibrium in genotype distribution was assessed using χ2 test. Following Kolmogorov–Smirnov test for normality, Mann–Whitney U test was used to evaluate age difference. The χ2 test was also used to assess the differences in gender between the PD cases and controls. The differences in genotype and allele frequencies were analyzed using logistic regression model with gender and age as covariates. Levels of gene expression were expressed as mean ± SE and analyzed by student’s *T*-test. A two-tailed *P* value < 0.05 was considered statistically significant.

## Results

### Identification of an antisense *Alu* insertion/deletion polymorphism in *ALDH1A1*

**Fig. 1:**
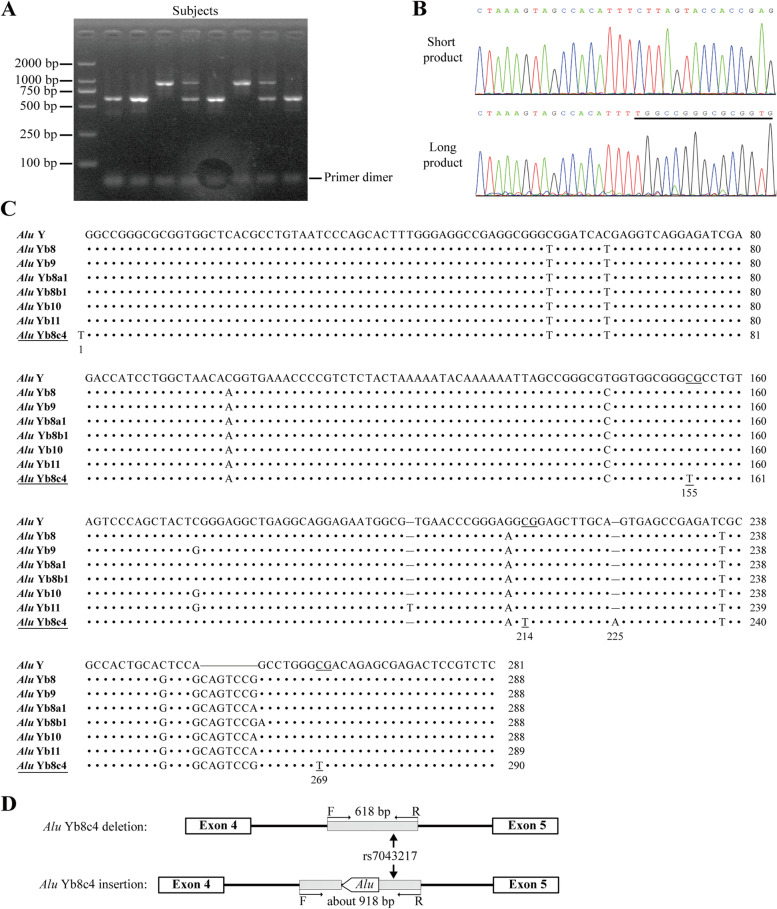
Identification of a novel antisense *Alu* element in intron 4 of *ALDH1A1*. **A** Agarose gel electrophoresis of PCR products amplified using the *Alu* primers. **B** Sequencing of the short and long PCR products. **C** Alignment of the additional insertion with *Alu* Y subfamilies. The identified variant was named *Alu* Yb8c4. **D** Schematic illustration of the *Alu* Yb8c4 insertion in *ALDH1A1 *

Amplifications using a primer pair targeting intron 4 of *ALDH1A1* resulted in an approximate 300 bp difference in the length of PCR products, suggesting a potential length polymorphism in this region (Fig. 1A; uncropped image in Additional file 1: Fig. S1). By sequencing both of the short and long PCR products, an additional segment was identified in the longer form, which was a 290 bp fragment appended with a poly-A tail at the 3'-end and located at 822 bp downstream of exon 4 of *ALDH1A1* (Fig. 1B; full sequencing results in Additional file 1: Fig. S2).

Multiple sequence alignments showed that the antisense sequence of this 290 bp fragment was highly homologous to the conserved *Alu* Y element, and appeared to be a variant of *Alu* Yb8 subfamily as featured by the GCAGTCCG insertion at position 254–261 (Fig. 1C). Compared to the Yb8 conserved region, this variant contained an extra T at the 1st position, an inserted A at the 225th position and three transition point mutations at the 155th (C > T), 214th (C > T), and 269th (C > T) positions. When the extra T at the 1st position and the poly-A segment were excluded, results of sequence alignment showed that the other four mutations within the Yb8 conserved region were shared by NC_000003.12 and NC_000008.11 (Additional file 1: Fig. S3). According to the standard nomenclature of *Alus* [20], we named this new *Alu* element as *Alu* Yb8c4, which is a new subclass of Yb8. A scheme of identifying the *Alu* polymorphism was illustrated in Fig. 1D.

### Association analyses of the *Alu* element of *ALDH1A1* with PD susceptibility

By using the cohort previously published [17], we found that the insertion/deletion polymorphism of the antisense Yb8c4 (asYb8c4) displayed a complete linkage disequilibrium (r^2^ = 1) with the PD risk SNP rs7043217. The deletion of asYb8c4 (asYb8c4^del^) was linked to the C allele of rs7043217, while the insertion of asYb8c4 (asYb8c4^ins^) was coupled with the T allele. The relative location of the asYb8c4 and rs7043217 was illustrated in Fig. 1D.

We thus collaboratively recruited an independent cohort consisting of 488 PD patients and 515 control subjects to validate the association of the asYb8c4 with PD, before performing further functional analyses. Genotype distributions of the asYb8c4 were in accordance with Hardy–Weinberg equilibrium (*P* > 0.05). The PD cases and controls were comparable in gender (*P* > 0.05) but different in age (*P* < 0.05). Results showed that genotype and allele frequencies of the asYb8c4 were significantly different between the cases and controls (*P* = 0.045 and* P* = 0.030, respectively), with the asYb8c4^ins^ serving as a risk allele for PD (OR = 1.224, 95% CI = 1.020–1.470; Table 1). The replication validated the association with PD discovered from the linked SNP, rs7043217 [17]. Further analysis of the asYb8c4^ins/del^ variants in three genetic models (additive, dominant, and recessive) showed that the asYb8c4 was significantly associated with PD in the recessive model (*P* = 0.016, OR = 1.520, 95% CI = 1.081–2.138) and additive model (*P* = 0.025, OR = 1.242, 95% CI = 1.028–1.502), again suggesting that the asYb8c4^ins^ was a risk allele for PD (Table 2).

**Table 1:** Genotype and allele frequencies of the asYb8c4 element in PD patients and controls

	**Genotype, n (%)**			***P***^a^	**Allele, n (%)**		***P***^a^	**OR (95% CI)**
	del/del	del/ins	ins/ins		del	ins		
Control	167 (34.2)	271 (52.6)	77 (15.0)	0.045*	605 (58.7)	425 (41.3)	0.030*	1.224 (1.020–1.470)
PD	135 (27.7)	256 (52.5)	97 (19.9)		526 (53.9)	450 (46.1)		

*CI* Confidence interval, *del* deletion, *ins* insertion, *OR* odds ratio, *PD* Parkinson’s disease

^**a**^Adjusted with age and sex

^*^P < 0.05

**Table 2:** Association between the asYb8c4 element and PD using dominant, recessive and additive models

Model	asYb8c4 genotype	*P*^a^	OR (95% CI)
Dominant	del/del *vs* del/ins + ins/ins	0.187	1.207 (0.913–1.597)
Recessive	del/del + del/ins *vs* ins/ins	0.016*	1.520 (1.081–2.138)
Additive	del/del *vs* del/ins *vs* ins/ins	0.025*	1.242 (1.028–1.502)

*CI* confidence interval, *del* deletion, *ins* insertion, *OR* odds ratio, *PD* Parkinson’s disease

^**a**^Adjusted with age and sex

^*^P < 0.05

### Functional impact of the asYb8c4 on *ALDH1A1* expression

**Fig. 2:**
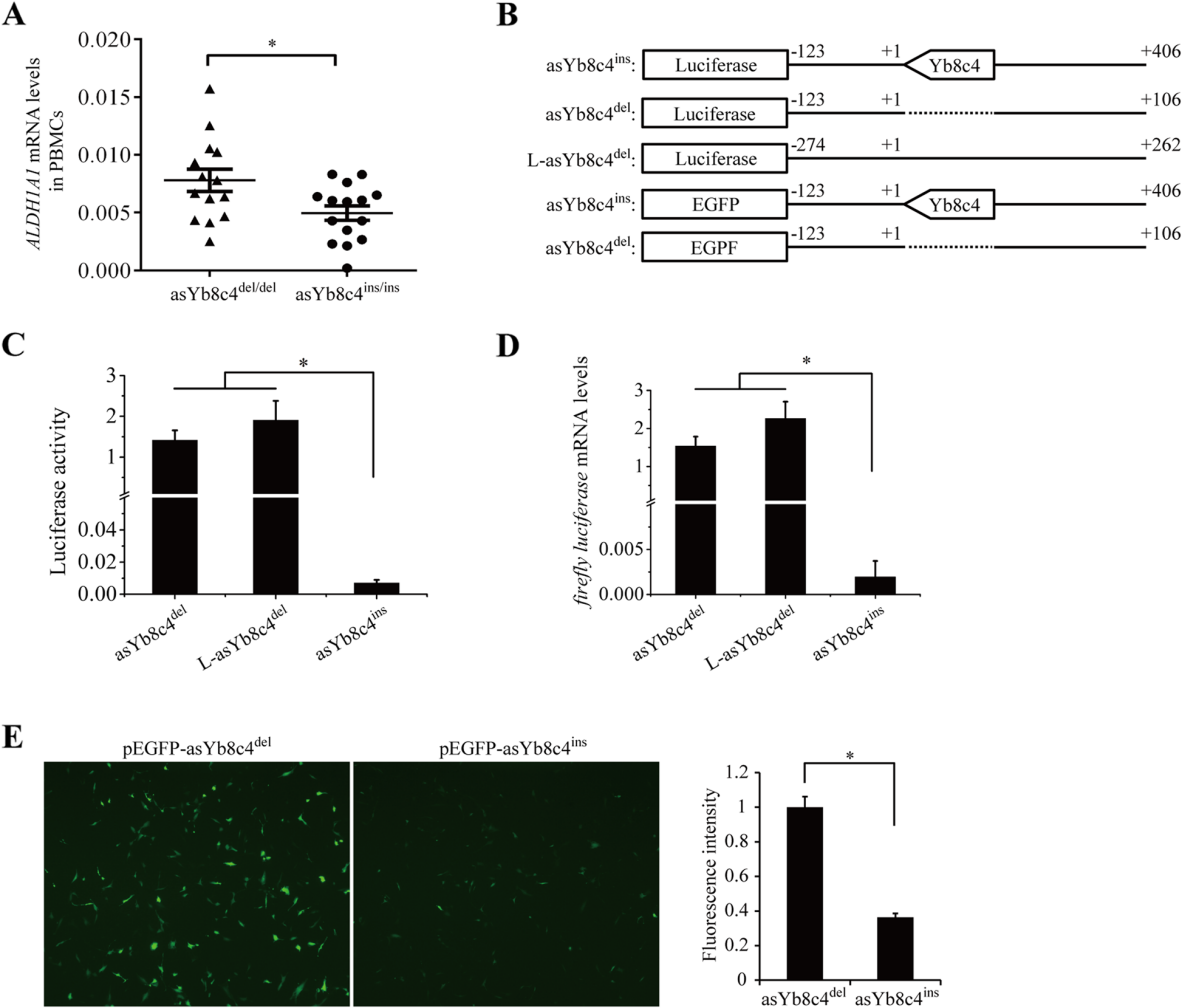
Impact of asYb8c4^ins^ and asYb8c4^del^ on gene expression. **A** mRNA expression of *ALDH1A1* in PBMCs of different genotype carriers (*n* = 14 for asYb8c4^del/del^; *n* = 15 for asYb8c4^ins/ins^). Results were normalized to their respective *ACTB* levels. **B** Schematic diagram of the luciferase reporter and EGFP constructs containing asYb8c4^ins^ (-123 to + 406), asYb8c4^del^ (-123 to + 106), and a long fragment with asYb8c4^del^ (L-asYb8c4^del^; -274 to + 262; length similar to asYb8c4^ins^). The position + 1 denotes the base immediately before asYb8c4^ins^. The indicated fragments were amplified from intron 4 of *ALDH1A1*. **C-D** Effect of asYb8c4^ins^, asYb8c4^del^ and L-asYb8c4^del^ on the firefly luciferase activity (C) and mRNA levels (D). Results were normalized to their respective Renilla luciferase activity or mRNA levels (*n* = 3). **E** Effect of asYb8c4^ins^ and asYb8c4^del^ on EGFP expression (*n* = 3). *, *P* < 0.05. *asYb8c4*^*del*^ antisense Yb8c4 deletion, *asYb8c4*^*ins*^ antisense Yb8c4 insertion, *PBMC* peripheral blood mononuclear cell

To determine the functional impact of asYb8c4^ins^ and asYb8c4^del^, mRNA expression levels of *ALDH1A1* were analyzed in PBMCs of 15 asYb8c4^ins/ins^ and 14 asYb8c4^del/del^ carriers with sex and age being matched (*P* > 0.05). *ACTB* (encoding β-actin) was used as a reference gene. Results of real-time PCR showed that mRNA expression of *ALDH1A1* was significantly (*P* = 0.02) lower in the asYb8c4^ins/ins^ group compared to that in the asYb8c4^del/del^ group (Fig. 2A). In addition, we analyzed mRNA expression levels of 3 reference genes, including *ACTB*, *GAPDH* and *HPRT1*, in 8 independent PBMC samples. *HPRT1* is also a gene commonly used for normalization in PBMCs [21, 22]. A highly similar expression pattern was observed within the three reference genes. The *ACTB* level showed a strong correlation with the mean level of *ACTB*, *GAPDH* and *HPRT1* (r^2^ = 0.9866; Additional file 1: Fig. S4), indicating that *ACTB* is an appropriate reference gene herein.

Luciferase reporter assays were employed to further confirm the role of asYb8c4 in gene expression. Three fragments were generated, including the asYb8c4^ins^ (529 bp), the asYb8c4^del^ (229 bp), and a long fragment of asYb8c4^del^ (named as L-asYb8c4^del^; 536 bp). The L-asYb8c4^del^ was designed as an additional control to match the asYb8c4^ins^ length to exclude potential effect caused by the length difference between the asYb8c4^ins^ and asYb8c4^del^. The three fragments were then respectively ligated into the 3’-untranlated region (UTR) of the luciferase gene (Fig. 2B). Results showed that the asYb8c4^ins^ insertion potently suppressed the expression of firefly luciferase as manifested by the activity assay (Fig. 2C) and the mRNA quantification (Fig. 2D), suggesting a transcriptional inhibition by asYb8c4^ins^. In contrast, the asYb8c4^del^ and L-asYb8c4^del^ insertions did not show a suppressive effect. Similar results were observed when asYb8c4^ins^ and asYb8c4^del^ fragments were incorporated into the 3’-UTR of pEGFP-C1. The EGFP expression was greatly reduced by the asYb8c4^ins^ (Fig. 2E).

### Potential mechanisms of the asYb8c4-mediated suppression of *ALDH1A1* expression

**Fig. 3:**
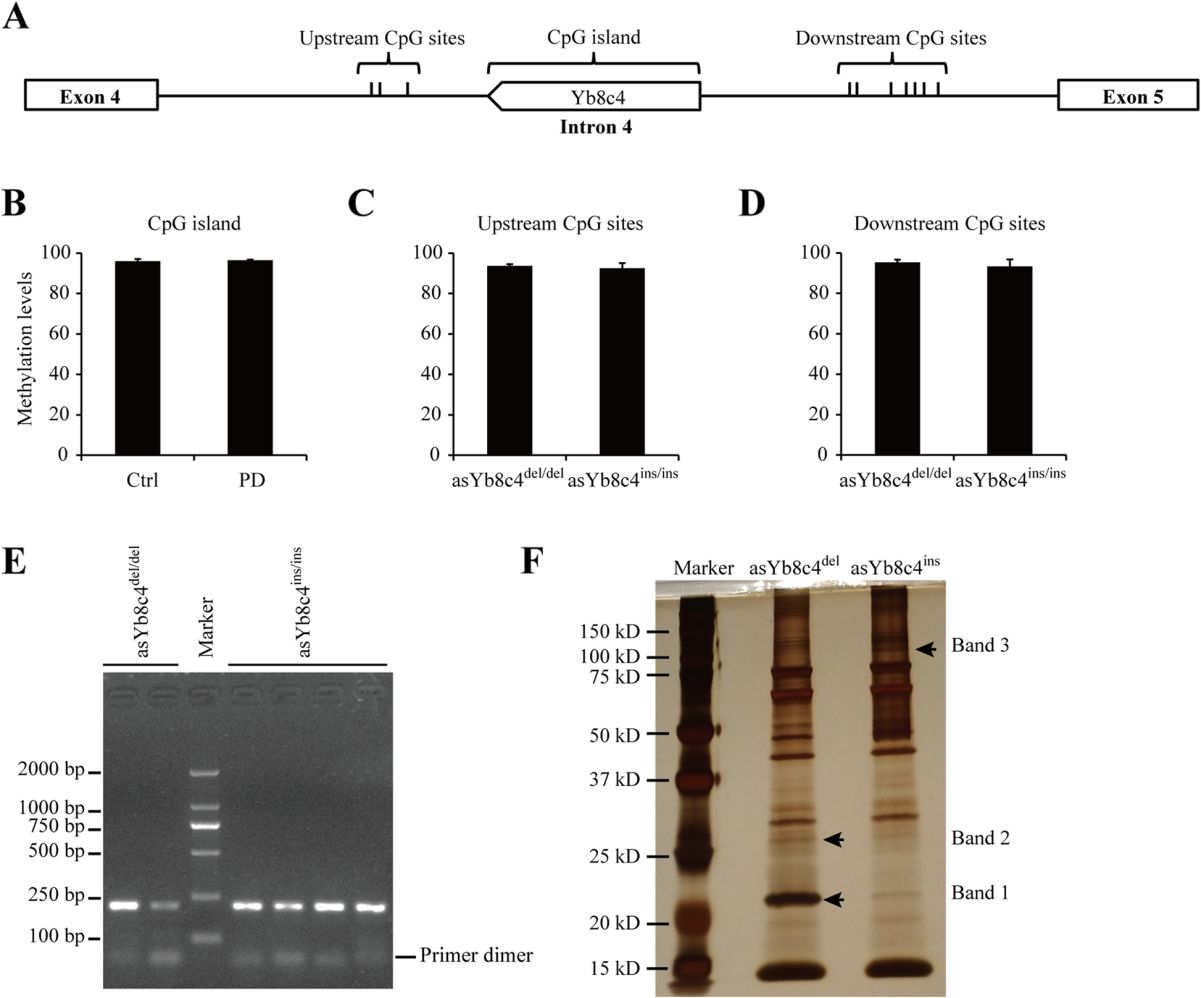
Potential mechanisms of the asYb8c4^ins^-mediated suppression of *ALDH1A1* expression. **A** Distribution schematic of the CpG island and sites in intron 4 of *ALDH1A1*. **B** Methylation levels of the asYb8c4^ins^-introduced CpG island in peripheral blood of PD cases and controls (*n* = 5). **C-D** Methylation levels of upstream (C) and downstream (D) CpG sites in peripheral blood of subjects carrying the asYb8c4^ins/ins^ or asYb8c4^del/del^ genotype (*n* = 5). **E** Reverse-transcribed PCR products amplified between *ALDH1A1* exons 3 and 5 from peripheral blood of subjects carrying the asYb8c4^ins/ins^ or asYb8c4^del/del^ genotype. **F** Silver staining gel of the DNA pull-down products from nuclear extract of SK-N-SH cells. Arrows indicate the protein bands used for mass-spectrometry identification. *asYb8c4*^*del*^ antisense Yb8c4 deletion, *asYb8c4*^*ins*^ antisense Yb8c4 insertion, *PD* Parkinson’s disease

Sequence analysis showed that the asYb8c4^ins^ introduced a CpG island of 23 CpG dinucleotides into intron 4 of *ALDH1A1* (Fig. 3A and Additional file 1: Fig. S5), of which 21 were located in the asYb8c4 element. Besides, there were 3 upstream CpG and 7 downstream CpG sites relative to the location of asYb8c4 (Fig. 3A and Additional file 1: Fig. S5). To determine whether these CpG sites modulated the inhibition of *ALDH1A1* expression by asYb8c4^ins^, cytosine methylation was analyzed in whole blood DNA. Results showed that the introduced CpG islands were comparably hypermethylated in PD cases (96.46%) and controls (95.98%) (Fig. 3B and Additional file 1: Fig. S6A). Both the upstream and downstream CpG sites were also similarly hypermethylated in two sets of control subjects respectively carrying the asYb8c4^ins/ins^ and asYb8c4^del/del^ genotypes (Fig. 3C-D and Additional file 1: Fig. S6B-C).

We then investigated whether the asYb8c4 insertion or deletion altered the RNA splicing pattern of *ALDH1A1*. Results of reverse-transcribed PCR between exons 3 and 5 showed a single band of the same size in subjects carrying the asYb8c4^ins/ins^ or asYb8c4^del/del^ genotype (Fig. 3E; uncropped image in Additional file 1: Fig. S1), excluding RNA splicing as a mechanism of such *ALDH1A1* under-expression.

A DNA pull-down assay combined with mass spectrometry was performed to identify potential transcription factors binding to the asYb8c4 insertion/deletion loci. Three unique bands eluted from the baits of asYb8c4^del^ (band 1 and 2) and asYb8c4^ins^ (band 3) were excised for proteomic analysis (Fig. 3F; uncropped image in Additional file 1: Fig. S1). Nucleus-localized proteins with molecular weights in the approximate range were as follows: 40S ribosomal protein S5, cilia- and flagella-associated protein 20, peroxiredoxin-1 and 60S ribosomal protein L9 from band 1 (20–23 kDa), serine/arginine-rich splicing factor 1 and 40S ribosomal protein S3 from band 2 (25–28 kDa), and pre-mRNA-processing factor 40 homolog A, DNA mismatch repair protein Msh2 and elongin-A from band 3 (85–115 kDa). Amongst, Msh2 and serine/arginine-rich splicing factor 1 were with higher unique peptide count. However, results of Gene Ontology suggested that no protein from the asYb8c4^del^ bait possessed molecular function of transcription activation, and none from the asYb8c4^ins^ bait possessed function of transcription repression (Additional file 1: Table S2).

## Discussion

A linkage between ALDH1 activity and PD pathogenesis is supported by evidence from animal studies and human results of brain expression [12, 13, 15]. In the present study, we disclose a novel antisense *Alu* element termed asYb8c4 in intron 4 of *ALDH1A1*. Its insertion/deletion polymorphism is genetically associated with susceptibility of PD in a Han Chinese population. The asYb8c4 appears to be a functional variant modulating *ALDH1A1* gene expression.

The association of asYb8c4 with PD in an independent cohort validates our previous finding that rs7043217 is a PD risk tag-SNP [17], since asYb8c4 is in complete linkage disequilibrium with rs7043217. The inheritance of rs7043217 was shown to be dominant and additive in the previous cohort, while the Yb8c4 was recessive and additive in the current one. The independent replication suggests that the inheritance of rs7043217 and Yb8c4 is most likely additive toward PD. As previously noted, we also found a genetic interaction between variants of *ALDH1A1* and *ALDH2* [17]. Indeed, the risk factors for PD are multifaceted [4]. Individual variants, genetic interactions in between, and functional impact on gene expressions, may collectively lead the way to PD pathogenesis. In addition, rs3764435 of *ALDH1A1* was reported to be associated with PD in Mexicans [23]. However, this SNP is not in linkage disequilibrium with rs7043217 in either Mexicans or Han Chinese as shown in 1000 Genomes Project.

*Alu* elements are the most abundant mobile elements and belong to the short interspersed elements with over 1 million copies in human genome [24]. The human Alu family is composed of a series of distinct subfamilies of different genetic ages that are characterized by some specific mutations, of which the Ya5 and Yb8 mainly represent the young subfamilies in humans [25]. Some of these young *Alu* elements can accumulate new mutations and generate new subfamilies. Subclasses of Yb elements, such as Yb8a1, Yb10 and Yb11, have been recently characterized suggesting that *Alu* elements are still evolving [26]. In this study, we find the three mutations of base substitution in Yb8c4 are all C > T transitions, which otherwise comprise CpG sites in Yb8. Indeed, cytosine methylation could lead to a high frequency of C to T transition mutation [27], indicating that the Yb8c4 is a younger subclass originated from Yb8. When searching Yb8c4 against the genome aggregation database (https://gnomad.broadinstitute.org; gnomAD), a multiallelic variant (13 segment insertion alleles, length 4–48 bases) is suggested at the same locus (chr9-72,928,069). No additional information is available yet regarding its frequency and presence in ethnicities. It remains to be determined whether these two polymorphisms are the same.

*Alu* element polymorphisms or mutations have been functionally linked to the risks of multiple diseases, including neurodegenerative disorders, haemophilia, breast cancer and heritable pulmonary arterial hypertension [28–30]. Amongst, Yb8 is one of the most active *Alu* subfamilies [31]. The current study showed that the asYb8c4 polymorphism in *ALDH1A1* is associated with the risk of PD, and the asYb8c4^ins^ may be a functional variant inhibiting gene expression at transcriptional level as manifested by our PBMC, EGFP and luciferase reporter results. These results suggest that rs7043217 and its represented SNPs [17] may functionally attribute their risk effect to the asYb8c4 polymorphism. As a comparison, little effect of inhibition was observed when an antisense *Alu* Sp element was ligated into the 3’-UTR of *EGFP* in a previous study using the same EGFP expression system [32]. These data suggest that Yb8c4 may be one of the functional subclasses of *Alu* elements.

Previous studies have shown that *Alu* elements can modulate gene transcription by introduction of transcription factor binding sites, change of methylation in their genomic vicinity, and alternation of RNA splicing and editing [33–35]. Unfortunately, no altered methylation status is detected in the Yb8c4-introduced CpG island between PD patients and controls, nor in its nearby CpG sites between asYb8c4^ins/ins^ and asYb8c4^del/del^ carriers. RNA splicing of *ALDH1A1* is also not changed by the insertion or deletion of asYb8c4. But we should also note that the expression, methylation, and splicing analyses were all performed on peripheral blood, which may differ from those in dopaminergic neurons. A further validation is desired if neuronal tissues are available. Among the nucleus-localized proteins acquired from the DNA pull-down assay, 40S ribosomal protein S5, 60S ribosomal protein L9 and 40S ribosomal protein S3 are component of the ribosome involved in translation process; Cilia- and flagella-associated protein 20 is involved in post-translational modification of the tubulin subunits of microtubules [36]; Peroxiredoxin-1 is an antioxidant enzyme [37]. Serine/arginine-rich splicing factor 1 and pre-mRNA-processing factor 40 homolog A are involved in pre-mRNA splicing [38, 39]; DNA mismatch repair protein Msh2 is involved in DNA repair [40]; Elongin-A is a transcription elongation factor increasing the overall rate of mRNA chain elongation by RNA polymerase II [41]. Since the asYb8c4 inhibits gene expression, the proper binding factor presumably should be transcription repressor for asYb8c4^ins^ allele and transcription activator for asYb8c4^del^ allele. However, no such protein factors are identified. Nonetheless, it is possible that the abundance of proper transcription factors may be under detection limit by this method. The underlying mechanism of asYb8c4 inhibiting gene transcription thus warrants further investigation. Of note, DNA mismatch repair protein Msh2 is of high possibility to bind asYb8c4^ins^ oligonucleotide, suggesting that this antisense *Alu* element is prone to DNA mismatch.

## Conclusions

The current study reveals an asYb8c4^ins/del^ polymorphism in intron 4 of *ALDH1A1* and demonstrates its positive association with PD susceptibility in a Han Chinese population. The asYb8c4^ins^ serves as a risk allele towards PD and may transcriptionally downregulate *ALDH1A1* gene expression. The asYb8c4 polymorphism may be a functional output of its linkage disequilibrium-linked SNPs. These findings genetically consolidate an involvement of ALDH1 in PD pathogenesis.

## Supplementary Information

**Additional file 1:** **Table S1.** Primers and restriction enzymes used in this study. **Table S2.** Mass-spectrometry results of DNA pulled-down proteins^a^. **Figure S1.** Uncropped gel images for Fig. 1A, Fig. 3E and Fig. 3F. The red boxes represent the cropped area. **Figure S2.** Sequencing results of the short product (A) and the long product (B). The insertion ended with poly-A is highlighted in yellow; The inserted position is after the T which is highlighted in green. **Figure S3.** Similarity search results of the Yb8c4 sequence excluding the T at the 1st position and poly-A segment. Checked box indicates 100% matched. **Figure S4.** Reference gene expressions in PBMC samples. (A) Ct values of ACTB, GAPDH and HPRT1 in individual samples. (B) Correlation analysis of gene expression between ACTB and mean of ACTB, GAPDH and HPRT1. n = 8. Ct, cycle threshold; PBMC, peripheral blood mononuclear cell. **Figure S5**. Distribution of CpG sites in intron 4 of ALDH1A1. The asYb8c4 element is indicated in red. The analyzed CpG sites are highlighted in yellow. **Figure S6.** Individual methylation plots of the asYb8c4ins-introduced CpG island (A), the upstream (B) and downstream (C) CpG sites. Closed cycle indicates methylated CpG, open circle indicates unmethylated CpG. asYb8c4del, antisense Yb8c4 deletion; asYb8c4ins, antisense Yb8c4 insertion; Ctrl, control; PD, Parkinson’s disease. 

## Data Availability

The sequence of *Alu* Yb8c4 has been deposited in GenBank under the accession number MW922677. The datasets used and/or analysed during the current study are available from the corresponding author on reasonable request.

## Abbreviations

ALDH: Aldehyde dehydrogenase
asYb8c4^del^: Antisense Yb8c4 deletion
asYb8c4^ins^: Antisense Yb8c4 insertion
CI: Confidence interval
DA: Dopaminergic
DOPAL: 3,4-Dihydroxyphenylacetaldehyde
LC–MS/MS: Liquid chromatography/tandem mass spectrometry
PBMC: Peripheral blood mononuclear cell
PCR: Polymerase chain reaction
PD: Parkinson’s disease
SNP: Single nucleotide polymorphism
