# Supplementary material for: An antisense Alu transposon insertion/deletion polymorphism of ALDH1A1 may functionally associate with Parkinson’s disease
Source: BMC Geriatr. 2022 May 16;22:427. doi: 10.1186/s12877-022-03132-1 (PMC9109383; doi:10.1186/s12877-022-03132-1)
Supplement: Supplementary file 1 — Additional file 1: Table S1. Primers and restriction enzymes used in this study. Table S2. Mass-spectrometry results of DNA pulled-down proteinsa. Figure S1. Uncropped gel images for Fig. 1A, Fig. 3E and Fig. 3F. The red boxes represent the cropped area. Figure S2. Sequencing results of the short product (A) and the long product (B). The insertion ended with poly-A is highlighted in yellow; The inserted position is after the T which is highlighted in green. Figure S3. Similarity search results of the Yb8c4 sequence excluding the T at the 1st position and poly-A segment. Checked box indicates 100% matched. Figure S4. Reference gene expressions in PBMC samples. (A) Ct values of ACTB, GAPDH and HPRT1 in individual samples. (B) Correlation analysis of gene expression between ACTB and mean of ACTB, GAPDH and HPRT1. n = 8. Ct, cycle threshold; PBMC, peripheral blood mononuclear cell. Figure S5. Distribution of CpG sites in intron 4 of ALDH1A1. The asYb8c4 element is indicated in red. The analyzed CpG sites are highlighted in yellow. Figure S6. Individual methylation plots of the asYb8c4ins-introduced CpG island (A), the upstream (B) and downstream (C) CpG sites. Closed cycle indicates methylated CpG, open circle indicates unmethylated CpG. asYb8c4del, antisense Yb8c4 deletion; asYb8c4ins, antisense Yb8c4 insertion; Ctrl, control; PD, Parkinson’s disease. [file 12877_2022_3132_MOESM1_ESM.pdf]

Table S1. Primers and restriction enzymes used in this study

Name		Primer
<u>Genotyping</u>		
Alu	F:	GAGTAATAAGCCTTTCCTACTGAC
	R:	GCAGCCTAAGTTAAGGGTTTGT
<u>Bisulfite sequencing PCR</u>		
CpG island	F:	AGTTGAGAGGAGAGTAAGAT
	R:	AGTGACTTCTCCCATTTTGT
upstream CpGs	F:	TTTAGTGTAGTTAGATTAGA
	R:	CATTATATACATACCCCACT
downstream	F:	AAGTTAGTAGGAAAGGTTTATTATTTATAG
CpGs	R:	ATAAACAAATATATTTAACATTCACAAAAT
<u>Real-time PCR</u>		
Firefly luciferase	F:	TGGTCGCTTCCGGATTGTTT
	R:	ACACCCGAGGGGGATGATAA
Renilla luciferase	F:	ACGGATGATAACTGGTCCGC
	R:	TAATACACCGCGCTACTGGC
ALDH1A1	F:	GCACGCCAGACTTACCTGTC
	R:	CCTCCTCAGTTGCAGGATTAAAG
β-actin	F:	TGGCACCCAGCACAATGAA
	R:	CTAAGTCATAGTCCGCCTAGAAGCA
<u>Reverse transcribed PCR</u>		
RNA splicing	F:	CGAAAGAGATCGTCTGCTGC
	R:	TGATTTGGCCACATACACCA
<u>Luciferase reporter constructs</u>		
asYb8c4$^{ins/del}$	F:	AGGGAGCTCTTTAGGAATACAATACCCT
	R:	ACAGAAGCTTCCTGGTTTCAGCA
L-asYb8c4^{del}	F:	AGGGAGCTCGTTTGTGTGTGTGAATCTGT
	R:	ACAGAAGCTTCAAATTCTCTAAATACATGA
<u>EGFP constructs</u>		
asYb8c4$^{ins/del}$	F:	AAGGCTCGAGTGATAAGGAATACAATACCCTCCAGG
	R:	AAGGCTGCAGGAAGCTTCCTGGTTTCAGCA
<u>DNA pull-down</u>		
DNA probes	F:	5'-Biotin-CGGAGTAAAGTAGGACAGTTCT
	R:	5'-Biotin-CAGGAGGTCAGAAGTGATAG

asYb8c4^{del}, antisense Yb8c4 deletion; asYb8c4^{ins}, antisense Yb8c4 insertion; F, forward; R, reverse.

Table S2. Mass-spectrometry results of DNA pulled-down proteins[a]

No.	Protein name	Unique peptide count	Molecular weight (kDa)	Molecular function (Gene Ontology)[b]
**Band 1 (20-23 kDa)**				
1	40S ribosomal protein S5	2	22.9	RNA binding; protein binding; structural constituent of ribosome
2	Cilia- and flagella-associated protein 20	1	22.8	RNA binding; protein binding
3	Peroxiredoxin-1	1	22.1	RNA binding; peroxidase activity; protein binding
4	60S ribosomal protein L9	1	21.9	rRNA binding; protein binding; structural constituent of ribosome
**Band 2 (25-28 kDa)**				
1	Serine/arginine-rich splicing factor 1	5	27.7	RNA binding; protein binding
2	40S ribosomal protein S3	2	26.7	RNA binding; protein binding; structural constituent of ribosome
**Band 3 (85-115 kDa)**				
1	Pre-mRNA-processing factor 40 homolog A	1	108.8	RNA binding; protein binding
2	DNA mismatch repair protein Msh2	6	104.7	DNA-binding; protein binding
3	Elongin-A	1	89.9	protein binding; transcription initiation from RNA polymerase II promoter

[a] Listed are nucleus-localized proteins with molecular weights in the approximate range of the bands as indicated in the parentheses.

[b] Listed are the most relevant.

Full gel of Fig. 1A

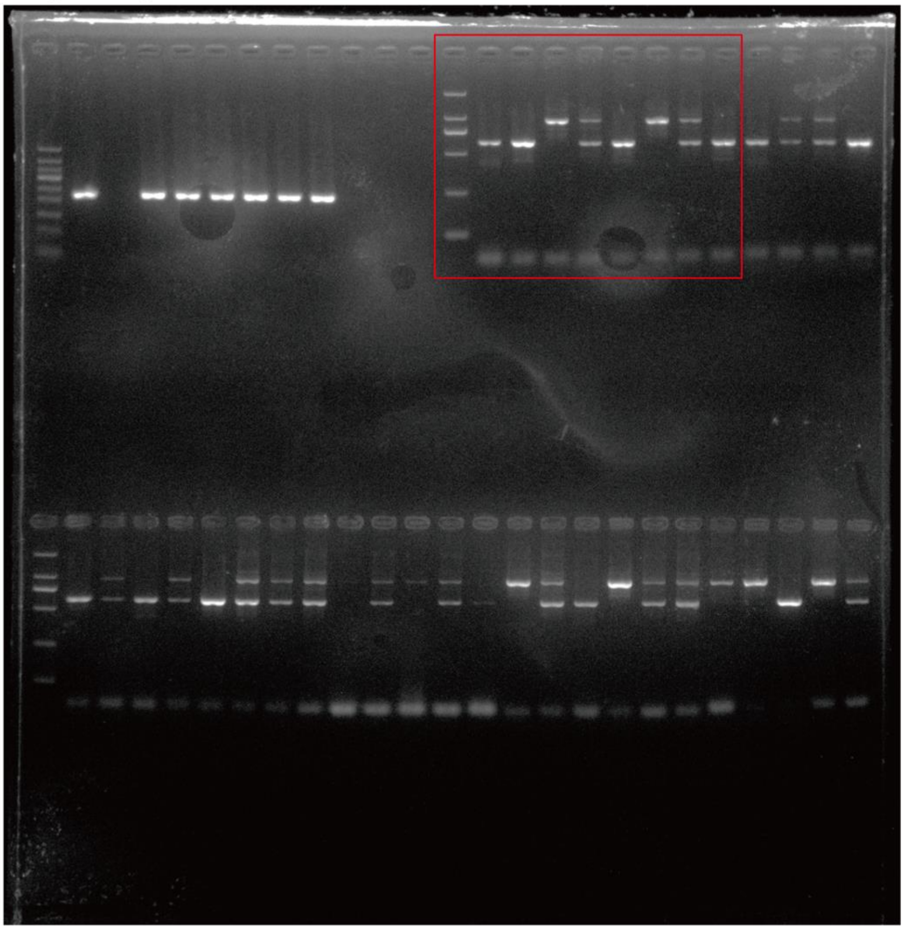

Full gel of Fig. 3E

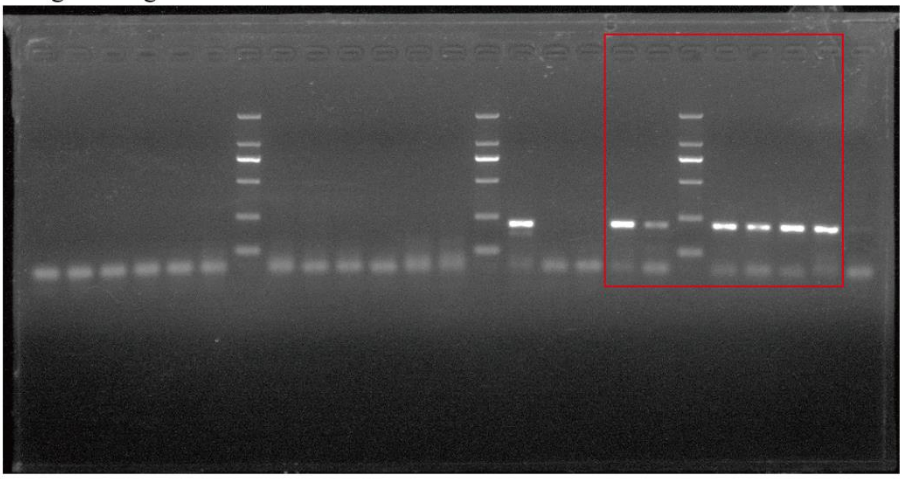

Full gel of Fig. 3F

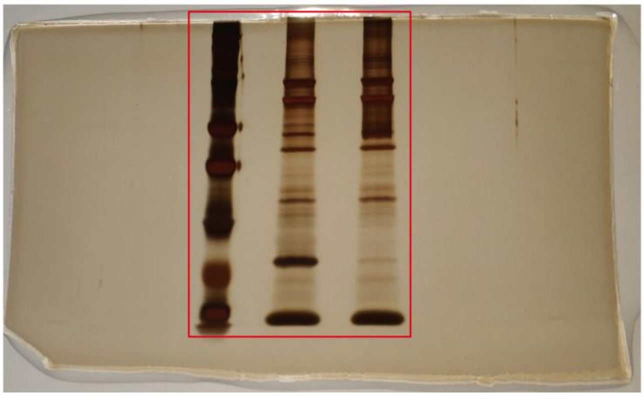

Fig. S1. Uncropped gel images for Fig. 1A, Fig. 3E and Fig. 3F. The red boxes represent the cropped area.

File: 103__J123-0404-217F_40286079528b839d015346ef3610605e_D06_1604000573G.ab1
Signal G:1694 A:1730 T:2164 C:1637
Sample: 01SH1604050487-S-D-A
592 bases in 7127 scans

Run Ended: Apr 5, 2016, 21:14:46
Comment:
Lane: 42 Base spacing 14.97
Page 1 of 2

A

10 20 30 40 50 60
G G T T T T T A A T G G A T T T T A T T T G C C T A C C A A A C A A T A A T A G C A A A T T C T C T A A A T A C A T G A A G A A A A T G A

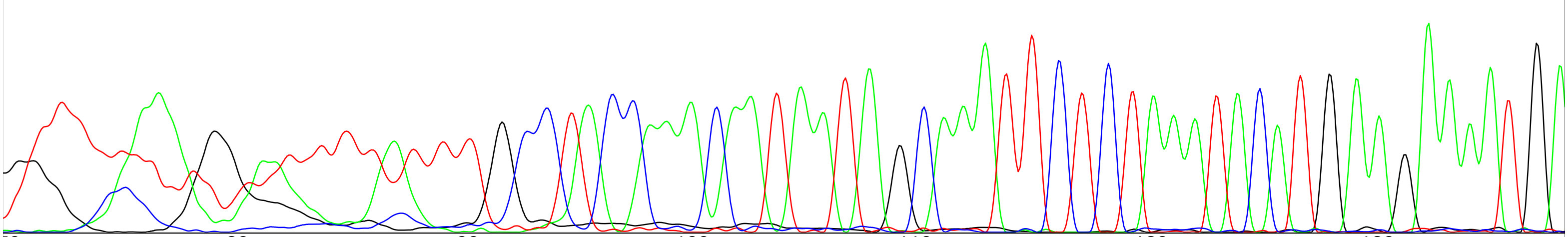

70 80 90 100 110 120 130
A C A G G A G T A T A A G A C A T T T A T T T T C A A T C T A A A T T T G A C C A A T T T A T G T G C C C C G A A G T T T A A T G T G G T

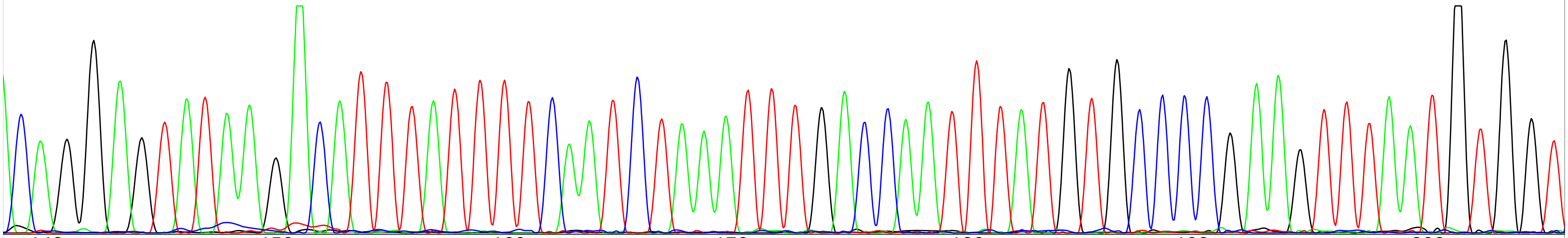

140 150 160 170 180 190 200
C A T C C A G A G A G A T A C A T T A T G C T T T A C A T T C A A T T C T G T T C T A G T T C C C A C A G A A G C T T C C T G G T T T C

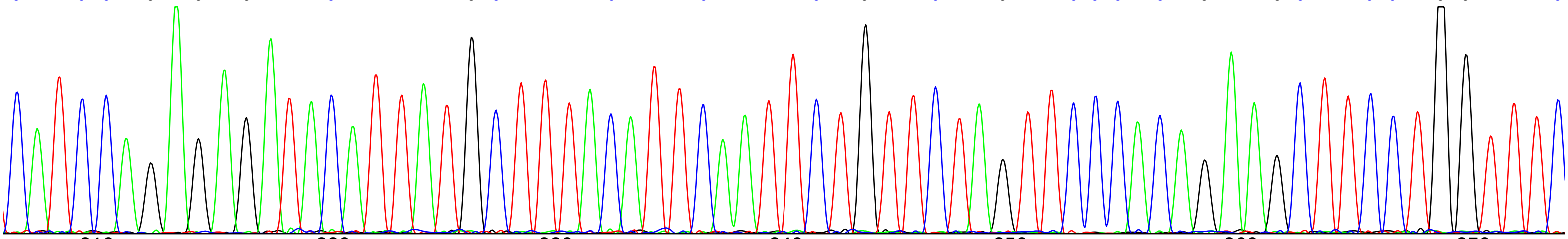

210 220 230 240 250 260 270
A G C A A G G A C T C T C T T C A T C A A G A T C T A T T T G T G T T T T A A G T G A C T T C T C C C A T T T T G T C C C G G A G T A A

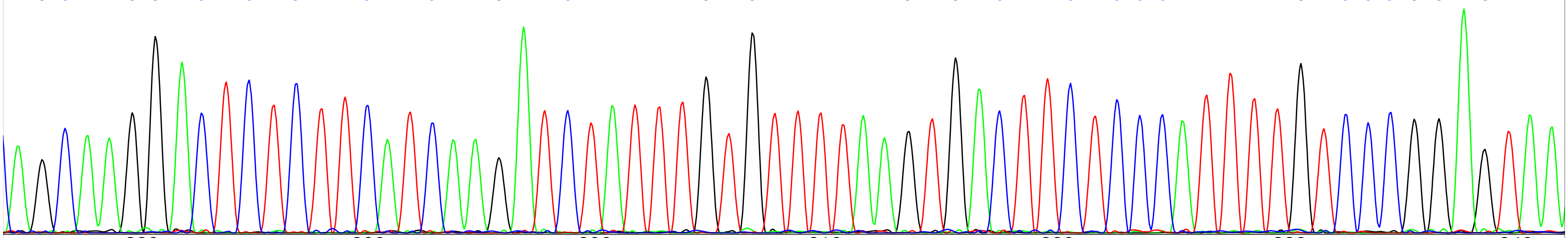

280 290 300 310 320 330 340
A G T A G G A C A G T T C T A A A G T A G C C A C A T T T C T T A G T A C C A C C G A G G T C T T A C T C T C C T C T C A A C T T T C T

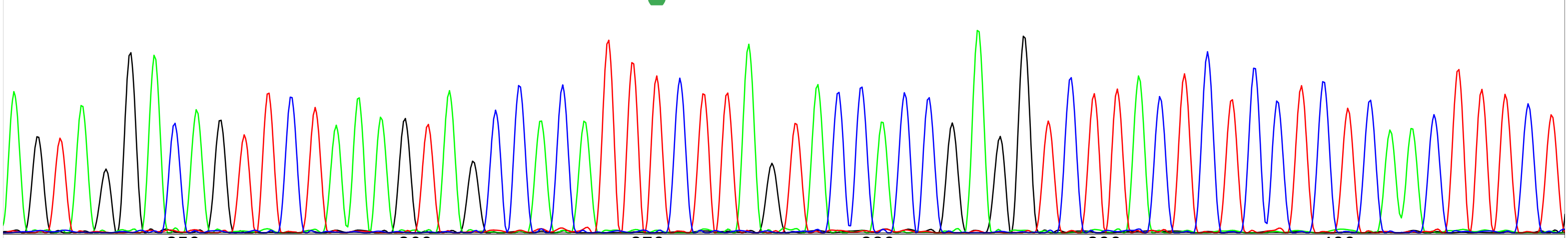

350 360 370 380 390 400
G C T A T C A C T T C T G A C C T C C T G C A T G C T C C A A T T G G A T T G G A A G T G C A C A T C A G G G C C A G T C C T G G A G G

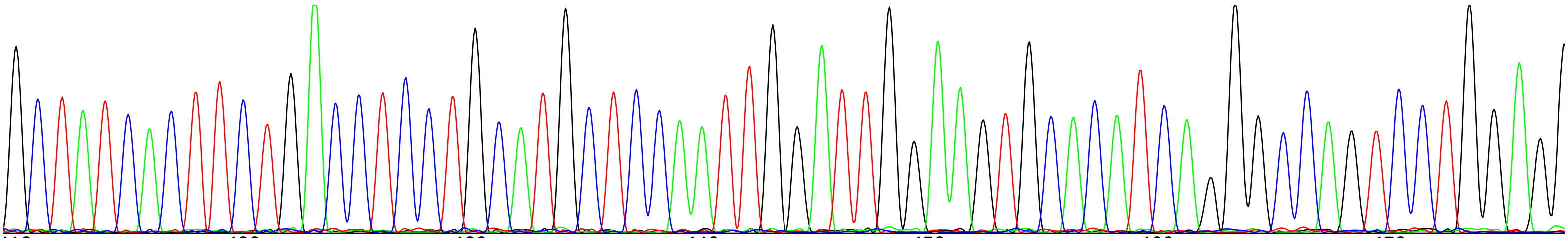

410 420 430 440 450 460 470
G G T A T T G T A T T C C T A A A G T T C A T A G G T T T G C C T G A A T T A A A T T C C A C C C A T A C T G T T T C C T A A A T G T G T

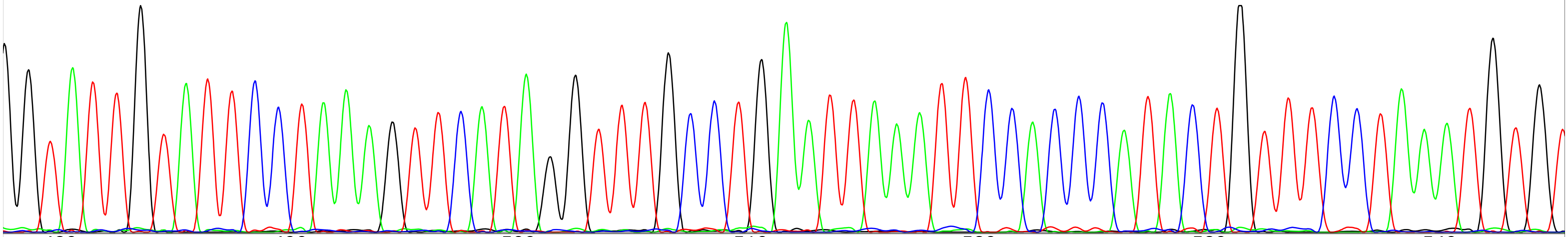

480 490 500 510 520 530 540
T G T G T T T T C A T G T T T C A C A T G A A A T A T C T T T A G T A T T T C T A C T C C A T T A T G T G C A T A C C C C A C T A A A A T

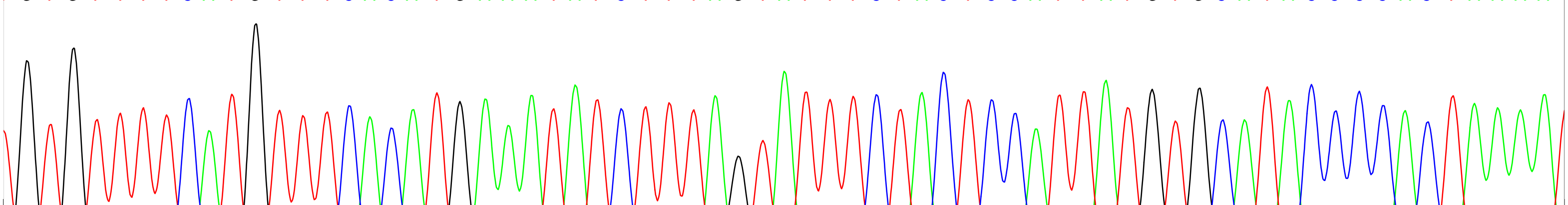

File: 103__J123-0404-217F_40286079528b839d015346ef3610605e_D06_1604000573G.ab1
Signal G:1694 A:1730 T:2164 C:1637
Sample: 01SH1604050487-S-D-A
592 bases in 7127 scans

Run Ended: Apr 5, 2016, 21:14:46
Comment:
Lane: 42 Base spacing 14.97

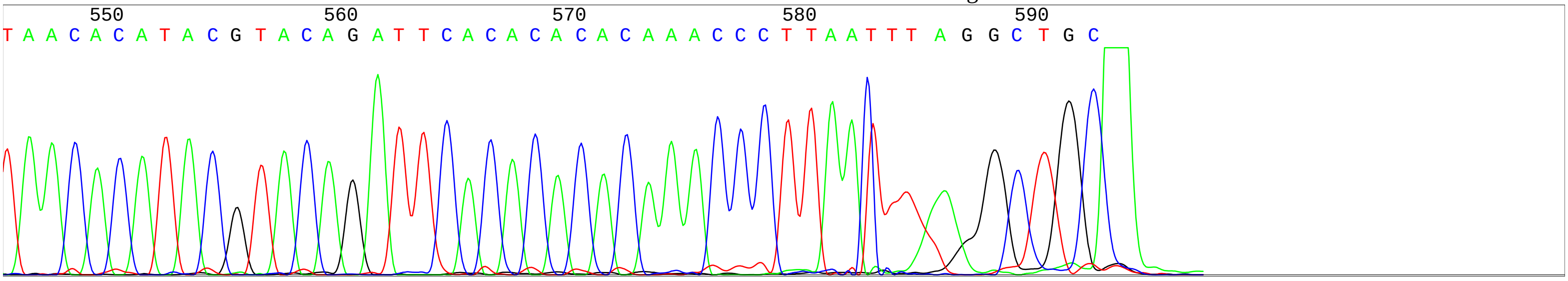

File: 105__J123-0404-217F_40286079528b839d015346ef3610605e_D05_1604000574Q.ab1
Signal G:273 A:243 T:247 C:175
Sample: 03SH1604050486-S-A-D
602 bases in 16217 scans

Run Ended: Apr 5, 2016, 21:35:28
Comment:
Lane: 13 Base spacing 12.77

B

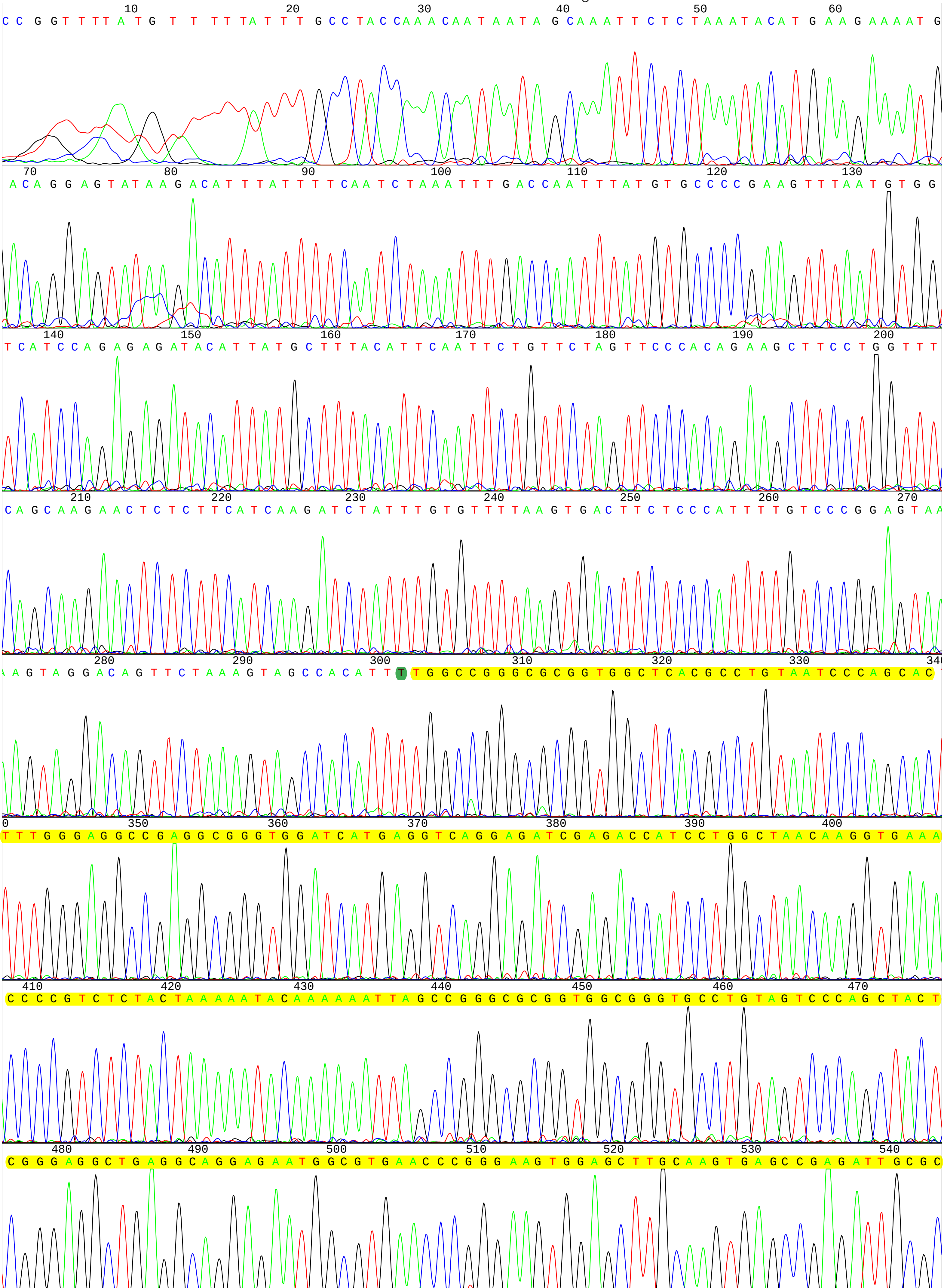

File: 105__J123-0404-217F_40286079528b839d015346ef3610605e_D05_1604000574Q.ab1
Signal G:273 A:243 T:247 C:175
Sample: 03SH1604050486-S-A-D
602 bases in 16217 scans

Run Ended: Apr 5, 2016, 21:35:28
Comment:
Lane: 13 Base spacing 12.77

550 560 570 580 590 600

C C A C T G C A G T C C G C A G T C C G G C C T G G G T G A C A G A G C G A G A C T C C G T C T C A A A A A A A A A A C

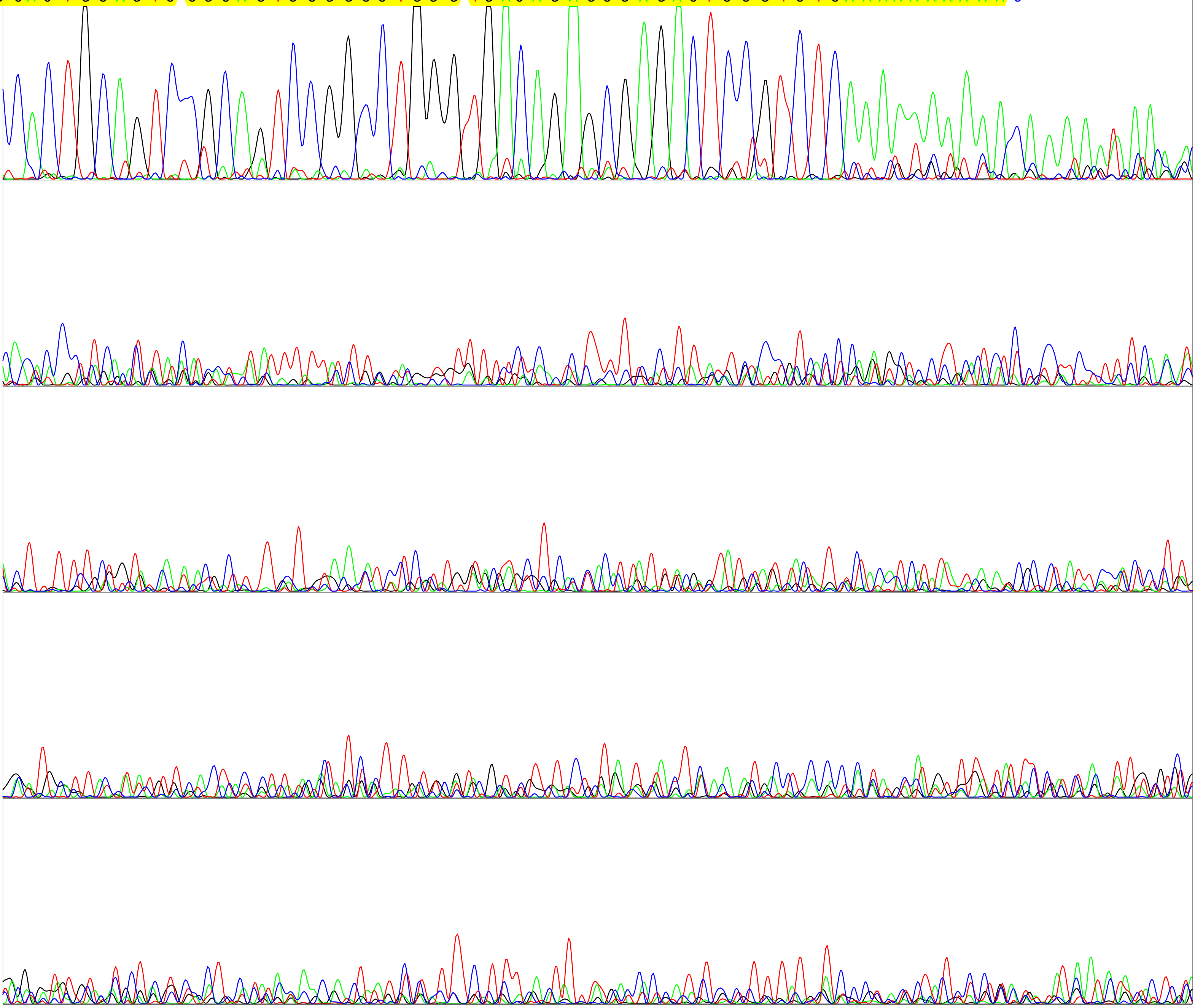

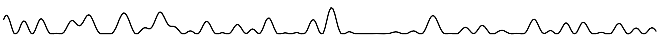

Fig. S2. Sequencing results of the short product (A) and the long product (B). The insertion ended with poly-A is highlighted in yellow; The inserted position is after the T which is highlighted in green.

	Description	Scientific Name	Common Name	Taxid	Max Score	Total Score	Query Cover	E value	Per. Ident	Acc. Len	Accession
☑	Homo sapiens chromosome 3, GRCh38.p13 Primary Assembly	Homo s...	human	9606	534	5.584e+06	100%	9e-150	100.00%	198295559	NC_000003.12
☑	Homo sapiens chromosome 8, GRCh38.p13 Primary Assembly	Homo s...	human	9606	534	3.764e+06	100%	9e-150	100.00%	145138636	NC_000008.11
☐	Homo sapiens chromosome 12, GRCh38.p13 Primary Assembly	Homo s...	human	9606	523	4.767e+06	100%	2e-146	99.31%	133275309	NC_000012.12
☐	Homo sapiens chromosome 1, GRCh38.p13 Primary Assembly	Homo s...	human	9606	518	8.424e+06	100%	9e-145	98.97%	248956422	NC_000001.11
☐	Homo sapiens chromosome 2, GRCh38.p13 Primary Assembly	Homo s...	human	9606	518	6.900e+06	100%	9e-145	98.97%	242193529	NC_000002.12
☐	Homo sapiens chromosome 11, GRCh38.p13 Primary Assembly	Homo s...	human	9606	518	3.976e+06	100%	9e-145	98.97%	135086622	NC_000011.10
☐	Homo sapiens chromosome 4, GRCh38.p13 Primary Assembly	Homo s...	human	9606	516	4.595e+06	100%	3e-144	98.96%	190214555	NC_000004.12
☐	Homo sapiens chromosome 5, GRCh38.p13 Primary Assembly	Homo s...	human	9606	516	4.839e+06	100%	3e-144	98.96%	181538259	NC_000005.10
☐	Homo sapiens chromosome 7, GRCh38.p13 Primary Assembly	Homo s...	human	9606	516	5.451e+06	100%	3e-144	98.96%	159345973	NC_000007.14
☐	Homo sapiens chromosome 9, GRCh38.p13 Primary Assembly	Homo s...	human	9606	516	4.198e+06	100%	3e-144	98.96%	138394717	NC_000009.12
☐	Homo sapiens chromosome 10, GRCh38.p13 Primary Assembly	Homo s...	human	9606	516	4.274e+06	100%	3e-144	98.96%	133797422	NC_000010.11
☐	Homo sapiens chromosome 13, GRCh38.p13 Primary Assembly	Homo s...	human	9606	516	2.493e+06	100%	3e-144	98.96%	114364328	NC_000013.11
☐	Homo sapiens chromosome 14, GRCh38.p13 Primary Assembly	Homo s...	human	9606	516	2.999e+06	100%	3e-144	98.96%	107043718	NC_000014.9
☐	Homo sapiens chromosome 15, GRCh38.p13 Primary Assembly	Homo s...	human	9606	516	3.223e+06	100%	3e-144	98.96%	101991189	NC_000015.10
☐	Homo sapiens chromosome 20, GRCh38.p13 Primary Assembly	Homo s...	human	9606	516	2.288e+06	100%	3e-144	98.96%	64444167	NC_000020.11
☐	Homo sapiens chromosome 6, GRCh38.p13 Primary Assembly	Homo s...	human	9606	512	4.927e+06	100%	4e-143	98.62%	170805979	NC_000006.12
☐	Homo sapiens chromosome 16, GRCh38.p13 Primary Assembly	Homo s...	human	9606	512	3.677e+06	100%	4e-143	98.62%	90338345	NC_000016.10
☐	Homo sapiens chromosome 18, GRCh38.p13 Primary Assembly	Homo s...	human	9606	512	2.006e+06	100%	4e-143	98.62%	80373285	NC_000018.10
☐	Homo sapiens chromosome X, GRCh38.p13 Primary Assembly	Homo s...	human	9606	512	4.228e+06	100%	4e-143	98.62%	156040895	NC_000023.11
☐	Homo sapiens chromosome Y, GRCh38.p13 Primary Assembly	Homo s...	human	9606	512	6.060e+05	100%	4e-143	98.62%	57227415	NC_000024.10

Fig. S3. Similarity search results of the Yb8c4 sequence excluding the T at the 1st position and poly-A segment. Checked box indicates 100% matched.

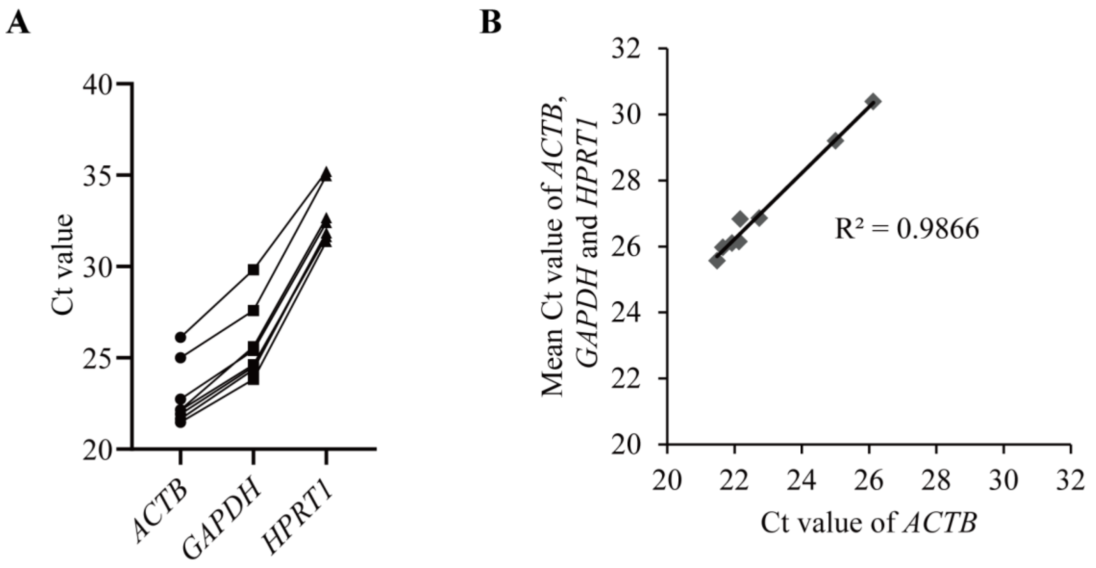

Fig. S4. Reference gene expressions in PBMC samples. (A) Ct values of *ACTB*, *GAPDH* and *HPRT1* in individual samples. (B) Correlation analysis of gene expression between *ACTB* and mean of *ACTB*, *GAPDH* and *HPRT1*. n = 8. Ct, cycle threshold; PBMC, peripheral blood mononuclear cell.

Upstream CpGs / CpG island / Downstream CpGs / Intron 4 of *ALDH1A1*

```
GTAAGTATCTTTGAGAAACCACTAATGGTGAGGATAGGAGCGAGGAGTTTACTATAGAGCTGAATAAT
TTCAAACTCTCCCTTTTAAAGATGTCAACCAAATAAGGCAAAATTATTTTCCTCTTGACTTTGAGACA
ACACAGTTTTCAACTTAGAAGTTCTATTAAAATTCATAAAAGGTCTTTTAAAGTTGTTTCAGCATAATC
ATGAAGATAACATTATAGATATTTTAGAAAATGTCAAAGTAAGAACATTCCTTTGGCAGTAATTACTGA
TCTGAGGCAATTGCCTCTCTAAGGTCCCAAACTTTTTAAAGCTGACCTCTGAAATTATTTATTTGGGTT
TTATTCCAATACTGACATTTATATCCTTTCTGTAATGTAATTATTTAAGCCTCTTTACATGTTCCAGTATA
AGTCAGTTATGGTCATCTGTGCCTGGTCAAACTCAGTGTAGTTAGACCAGACTAATCAAACAAGCCA
GTTCACACGGACTCGTTTCAAATATCTTCAAAGCAAGATGGGACTGTGTTTGCAGCCTAAGTTAAGG
GTTTGTGTGTGTGAATCTGTACGTATGTGTTATTTTAGTGGGGTATGCACATAATGGAGTAGAAATACT
AAAGATATTTCATGTGAAACATGAAAACACACACATTTAGGAAACAGTATGGGTGGAATTTAATTCAG
GCAAACCTATGAACTTTAGGAATACAATACCCTCCAGGACTGGCCCTGATGTGCACTTCCAATCCAAT
TGGAGCATGCAGGAGGTCAGAAGTGATAGCAGAAAGTTGAGAGGAGAGTAAGACCTCGGTGGTACT
AAGAAATGTGGCTACTTTATTTTTTTTTTGAGACGGAGTCTCGCTCTGTCACCCAGGCCGGACTGCGG
ACTGCAGTGGCGCAATCTCGGCTCACTTGCAAGCTCCACTTCCCGGGTTCACGCCATTCTCCTGCCTC
AGCCTCCCGAGTAGCTGGGACTACAGGCACCCGCCACCGCGCCCGGCTAATTTTTTGTATTTTTAGTA
GAGACGGGGTTTCACCTTGTTAGCCAGGATGGTCTCGATCTCCTGACCTCATGATCCACCCGCCTCGG
CCTCCCAAAGTGCTGGGATTACAGGCGTGAGCCACCGCGCCCGGCCAAAATGTGGCTACTTTAGAAC
TGTCCTACTTTACTCCGGGACAAAATGGGAGAAGTCACTTAAAACACAAATAGATCTTGATGAAGAG
AGTTCTTGCTGAAACCAGGAAGCTTCTGTGGGAACTAGAACAGAATTGAATGTAAAGCATAATGTAT
CTCTCTGGATGACCACATTAAACTTCGGGGCACATAAATTGGTCAAATTTAGATTGAAAATAAATGTC
TTATACTCCTGTCATTTTCTTCATGTATTTAGAGAATTTGCTATTATTGTTTGGTAGGCAAATAAAATACA
TTTTAGAACAAGTCAGTAGGAAAGGCTTATTACTCATAGGGAGATTTTCTTTTTAATTCTACTGTTTTT
AATTTTACTATTTCTAAAAATTATATTATAACTTTTAAAAAGAATTCTCTTATTGGTAGAACTTTGAGCT
ACCAGAAACAAATTGGAATGTGGTTCTTCTCTTCGCCAGAGACCTCCTACTTCCTTTCTCGTCCCTTT
TTTATAACACTTAGAGAATAAAGATAATTTTCAAATCAGGCCTCCTAGAAGATGAAAGGTGGAATTAA
TTATCTTTCAGATTTTTGCCAATTTTGCCTCTTTGATTTCACCTAACTCAATTTTATGGTTCCTATCATTT
CGAATTACTATTTTAGGGCTTCACTTACCATTTTGGGAATAGTTGGAGATATAGATGGTCTCGATCTCTT
GACCTCGTGATCTGCCTGCCTCGGCCTCCCAAAGTGCTGGGATTACAGGCGTGAGCCACCACACCTG
GCCAGATATATGTTTTTAAAATTATTGTCTACACCTTTCTCTCACATCACCAACCACCATTTTGTGAATG
TCAAATACATTTGTTTATATATGATTGTGTGTGTTTTATTTTTCAGA
```

Fig. S5. Distribution of CpG sites in intron 4 of *ALDH1A1*. The asYb8c4 element is indicated in red. The analyzed CpG sites are highlighted in yellow.

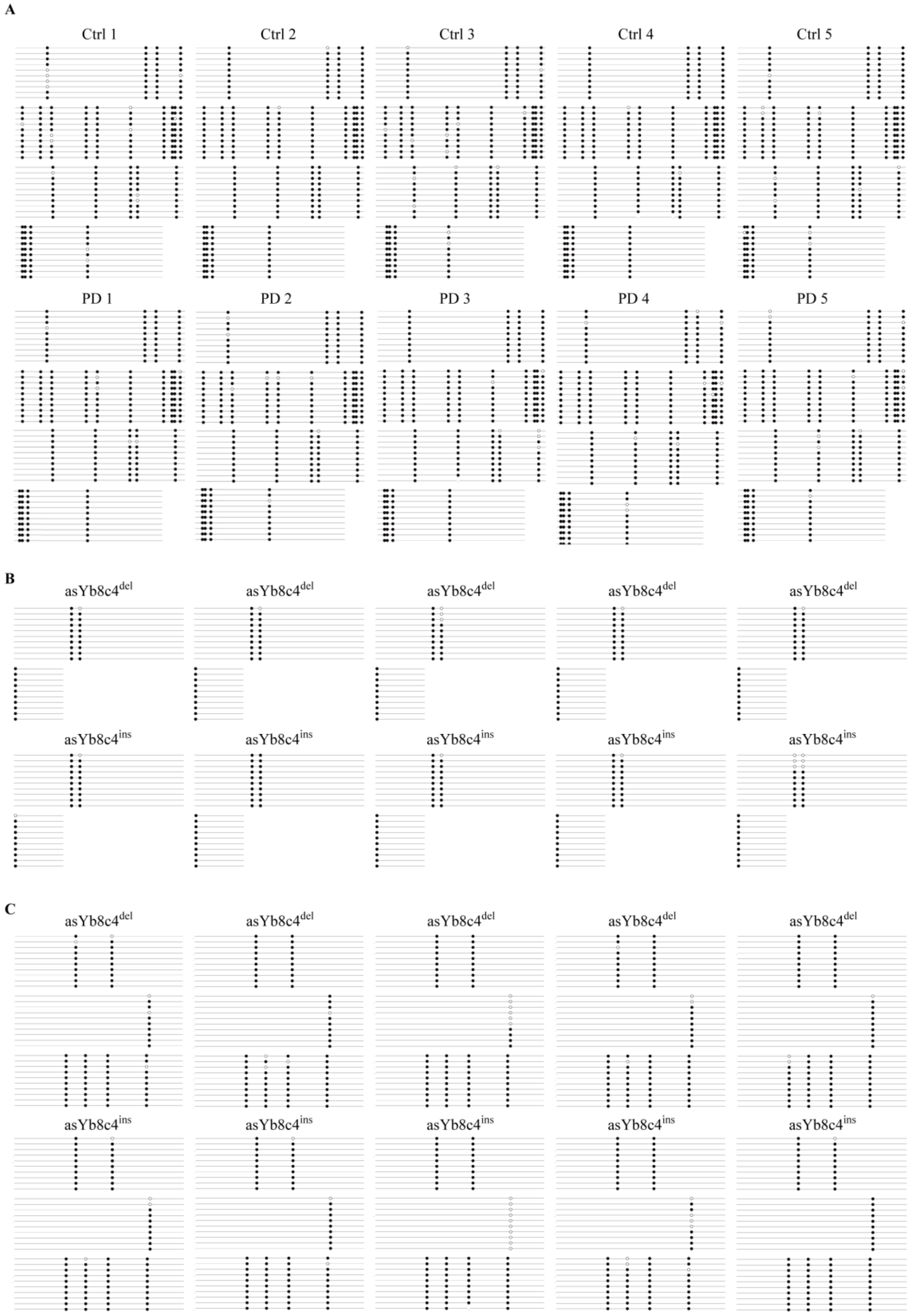

Fig. S6. Individual methylation plots of the asYb8c4^{ins}-introduced CpG island (A), the upstream (B) and downstream (C) CpG sites. Closed cycle indicates methylated CpG, open circle indicates unmethylated CpG. asYb8c4^{del}, antisense Yb8c4 deletion; asYb8c4^{ins}, antisense Yb8c4 insertion; Ctrl, control; PD, Parkinson's disease.
